# Fasciite nécrosante: une redoutable affection!

**DOI:** 10.11604/pamj.2017.26.6.10859

**Published:** 2017-01-04

**Authors:** Sara Elloudi, Fatima Zahra Mernissi

**Affiliations:** 1 Service de Dermatologie et Vénérologie, Hôpital Universitaire Hassan II, Fès, Maroc

**Keywords:** Diabète, bulles hémorragiques, choc septique, Diabetes, hemorrhagic bullae, septic shock

## Image en médecine

La fasciite nécrosante est une infection bactérienne touchant le derme profond, l’hypoderme et l’aponévrose superficielle. C’est une infection sévère mortelle dans 30% des cas. Une association polymicrobienne est fréquemment notée. Les facteurs associés sont le diabète, l’alcoolisme chronique, la toxicomanie intraveineuse, et l’immunosuppression. Nous rapportons le cas clinique d’un patient diabétique âgé de 65 ans qui était admis aux urgences pour un état de choc septique et dont l’examen dermatologique objectivait des placards œdémateux froids et livédoides surmontées de phlyctènes hémorragiques intéressant le membre supérieur et inférieur droits, faisant évoquer des fasciites nécrosantes, le patient a installé rapidement une défaillance multi-viscérale occasionnant son décès. Il est capital de reconnaître au plus vite la fasciite nécrosante. En effet, la précocité de la mise en œuvre du traitement médicochirurgical conditionne le pronostic de cetteinfection redoutable qui peut conduire au décès.

**Figure 1:**
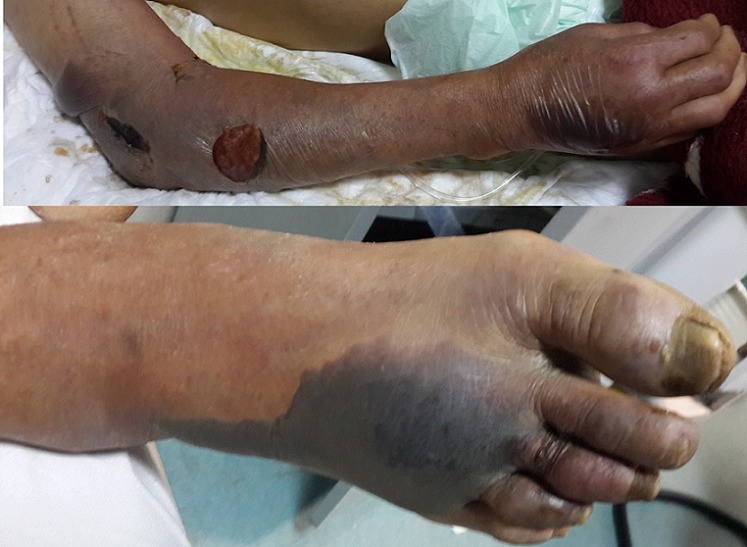
Placards œdémateux et phlyctènes hémorragiques du membre supérieur et inférieur droits

